# Aryl hydrocarbon receptor-mediated potencies in field-deployed plastics vary by type of polymer

**DOI:** 10.1007/s11356-019-04281-4

**Published:** 2019-02-04

**Authors:** Christine Schönlau, Maria Larsson, Monika M. Lam, Magnus Engwall, John P. Giesy, Chelsea Rochman, Anna Kärrman

**Affiliations:** 1 MTM Research Centre, School of Science and Technology, Örebro University, Örebro, Sweden; 2 Department of Veterinary Biomedical Sciences and Toxicology Centre, University of Saskatchewan, Saskatoon, Saskatchewan Canada; 3 University of California, Davis, 1089 Veterinary Medicine Dr, Davis, CA 95616 USA; 4 Department of Ecology and Evolutionary Biology, University of Toronto, Toronto, Canada

**Keywords:** Microplastics, In vitro bioassays, Ah receptor, PAH, H4IIE-*luc*

## Abstract

Plastic is able to sorb environmental pollutants from ambient water and might act as a vector for these pollutants to marine organisms. The potential toxicological effects of plastic-sorbed pollutants in marine organisms have not been thoroughly assessed. In this study, organic extracts from four types of plastic deployed for 9 or 12 months in San Diego Bay, California, were examined for their potential to activate the aryl hydrocarbon receptor (AhR) pathway by use of the H4IIE-*luc* assay. Polycyclic aromatic hydrocarbons (PAH), including the 16 priority PAHs, were quantified. The AhR-mediated potency in the deployed plastic samples, calculated as bio-TEQ values, ranged from 2.7 pg/g in polyethylene terephthalate (PET) to 277 pg/g in low-density polyethylene (LDPE). Concentrations of the sum of 24 PAHs in the deployed samples ranged from 4.6 to 1068 ng/g. By use of relative potency factors (REP), a potency balance between the biological effect (bio-TEQs) and the targeted PAHs (chem-TEQs) was calculated to 24–170%. The study reports, for the first time, in vitro AhR-mediated potencies for different deployed plastics, of which LDPE elicited the greatest concentration of bio-TEQs followed by polypropylene (PP), PET, and polyvinylchloride (PVC).

**Electronic supplementary material:**

The online version of this article (10.1007/s11356-019-04281-4) contains supplementary material, which is available to authorized users.

## Introduction

In the early 1970s, plastic particles were detected in marine systems for the first time (Carpenter and Smith 1972). By the late 1990s, more than 180 marine species were recorded to demonstrate ingested plastic particles (Laist 1997). Today, three quarters of all marine debris is plastic, and more than 500 marine and coastal species, including fish, seabirds, marine mammals, marine reptiles, and brackish turtles, are known to be affected by marine debris via ingestion or entanglement (CBD 2016). Considering the increasing global plastic consumption per year, numbers of affected species can be expected to increase. In 2015, worldwide production of plastic was 322 million tonnes, of which the European Union contributed 58 million tonnes (PlasticsEurope 2016). It has been estimated that at least 8 million tonnes of plastics enter the oceans every year from land-based sources (Jambeck et al. 2015).

While the mere physical threat such as entanglement, strangulation, and abrasion that plastic can pose to organisms is obvious, it has also been hypothesized that plastics hold a toxicological risk due to the transfer of pollutants when the particles are ingested (Teuten et al. 2007; Teuten et al. 2009). Numerous studies have demonstrated that a variety of anthropogenic environmental pollutants can be found on different types of marine and beached plastic debris (GESAMP 2016). Additionally, there is mounting evidence for the presence of plastic particles in higher trophic level organisms (CBD 2016) as well as in commercially important fish and shellfish species (Romeo et al. 2015; Van Cauwenberghe and Janssen 2014). Trophic transfer of microplastics has also been observed in laboratory studies (Farrell and Nelson 2013; Watts et al. 2014). Besides a possible bioaccumulation of contaminants due to ingested plastics (Teuten et al. 2009), there might be a potential for biomagnification of contaminants throughout the food chain. In some cases, a trophic transfer of contaminants can lead to a biomagnification, this is generally said to happen when the trophic transfer factor (TTF) is greater than 1. The TTF is defined as the concentration of contaminant in consumer tissue divided by the concentration of the contaminant in food (Suedel et al. 1994).

The sorption of chemicals from ambient environments to plastic is influenced by the physical and chemical properties of the type of polymer (Karapanagioti and Klontza 2008; Rochman et al. 2013; Teuten et al. 2007), as well as the surrounding environment (Velzeboer et al. 2014). Furthermore, sorption of chemicals is compound-specific and reflected in varying partition coefficients (Ziccardi et al. 2016). For instance, sorption affinities increase with increasing hydrophobicity (Smedes et al. 2009), while sorption kinetics decrease with increasing hydrophobicity (Endo et al. 2013; Rochman et al. 2013). In general, rubbery polymers such as low-density polyethylene (LDPE) and polypropylene (PP) are expected to have a greater diffusivity than glassy polymers like polyethylene terephthalate (PET) and polyvinylchloride (PVC) (Rochman et al. 2013; Teuten et al. 2009). Due to its great sorptive capacity for hydrophobic organic compounds (HOCs), polyethylene (PE) has been widely used in passive sampling approaches in different media (Zabiegala et al. 2010) and it is well known that there is a good correlation between polyethylene-water partition coefficients (log K_PE-W_) and octanol-water partition coefficients (log K_OW_) for different HOCs (Adams et al. 2007; Choi et al. 2013). In the marine environment, PE, PP, and polystyrene (PS) are the three most commonly observed types of polymers (Hidalgo-Ruz et al. 2012). A variety of anthropogenic chemicals including polychlorinated biphenyls (PCBs), polycyclic aromatic hydrocarbons (PAHs), polybrominated biphenyl ethers (PBDEs) and organochlorine pesticides (OCPs) can be associated with marine plastics. Within these groups of aforementioned HOCs, some chemicals are potentially harmful to humans and wildlife, and can act as mutagens, carcinogens, and endocrine disruptors or cause oxidative stress (Costa et al. 2008; Fonnum et al. 2006; Fry 1995; Zhang et al. 2016). Doubts have been raised about the importance of marine plastics as a vector to transfer potentially hazardous HOCs to organisms upon ingestion, because other sources such as natural prey and marine components, like colloids and sediments, in general hold greater concentrations of HOCs compared to plastics, and therefore potentially predominate the flux of HOCs to marine organisms (Herzke et al. 2016; Koelmans et al. 2016). Other researchers have suggested that the transfer of additives, such as flame retardants and plasticizers, from plastics, could be of greater importance than the transfer of sorbed HOCs, such as PCBs (Rochman et al. 2014; Tanaka et al. 2013; Teuten et al. 2009). Nevertheless, there is limited experimental data available on biomarker of exposure responses on cellular and subcellular levels upon exposure to microplastics containing adsorbed HOCs (Ziccardi et al. 2016), and it is important to understand how the mixture of plastics and sorbed HOCs will interact with biota.

The objectives of this study were to assess the potentials of four types of primary plastics, also referred to as preproduction or virgin plastics, to activate the aryl hydrocarbon receptor (AhR), by use of the H4IIE-*luc*, in vitro, transactivation assay. The plastic pellets were previously deployed in an urban marine environment (Rochman et al. 2013), and thus contained sorbed HOCs. Simultaneously, contribution of PAHs to AhR-mediated signal transduction was assessed by use of a potency balance analysis. In vitro bioassays are useful to screen for integrated effects of contamination in a variety of matrices (Eichbaum et al. 2014). One benefit of in vitro assays, based on expression of specific reporter genes, is that they integrate the overall potency of a mixture to modulate specific biological activities of an environmental sample as well as correct for interactions between and among constituents and correct for chemical potencies. The herein utilized H4IIE-*luc* assay (Murk et al. 1996) has been applied as a biomarker to screen for dioxin-like chemicals (DLCs) including co-planar non-di-ortho-substituted polychlorinated biphenyls (DL-PCBs) and some PAHs (Giesy and Kannan 1998; Larsson et al. 2012; Van den Berg et al. 2006). The cytosolic receptor protein, AhR, mediates most of the toxicological effects of specific halogenated aromatic hydrocarbons (HAH) and is also involved in inducing transcription of certain enzymes that catalyze metabolic activation of PAHs to mutagenic derivatives (Hankinson 1995). These effects can include impaired neurobehavioral functions and reproductive and neurochemical alterations such as reduced sperm production and reduced thyroid hormone levels in the brain and body (Brouwer et al. 1995). The Ah receptor evolved very early in the evolution of vertebrates, and thus, exists in a variety of species including fishes (Hankinson 1995).

In a long-term field study, exposing preproduction plastic pellets to the marine system in the San Diego Bay, it was observed that polyethylene plastics sorbed greater amounts of PCBs and PAHs than did PP, PET, and PVC (Rochman et al. 2013). Therefore, it was hypothesized by the authors that PE plastics might pose a greater toxicological risk to organisms when ingested than other polymer types. In the present study, a subset of the samples that were analyzed by Rochman et al. (2013) was used to assess the hypothesis that extracts of PE plastics will elicit a greater AhR-mediated response than PP, PET, and PVC. A potency balance was conducted using concentrations of 42 polycyclic aromatic compounds (PACs) since the previous study demonstrated that PAHs (*n* = 15) were the predominant AhR agonists found in the samples.

## Materials and methods

### Chemicals

Dimethyl sulfoxide (DMSO) (99.9%) was purchased from Sigma Aldrich (Stockholm, Sweden) and n-hexane (≥ 98%) was purchased from VWR (Stockholm, Sweden). Steady Lite plus™ was purchased from Perkin Elmer (Hägersten, Sweden). The 2,3,7,8-tetrachlorodibenzo-*p*-dioxin (TCDD) standard, with a purity of 99.1%, was from AccuStandard Inc. (New Haven, USA).

For more information regarding chemical standards, refer to the Supporting Information (SI).

### Deployment

Four types of plastic preproduction pellets, PP, PVC, PET, and LDPE, were deployed for 9 and 12 months from docks at three locations (Figure S1 of the Supporting Information), Shelter Island (SI), Harbor Excursion (HE), and Nimitz Marine Facility (NMF), in the San Diego Bay, CA, USA. Additionally, one blank sample of every polymer type was analyzed before deployment, time zero. Hereafter, samples are named by the polymer acronym, acronym of the site of deployment and number of the months of deployment. PET samples were cylindrical (2 mm diameter, 3 mm long), pellets of LDPE, PP‚ and PVC were spherical (3 mm diameter). All docks were floating except for the Nimitz Marine Facility, which was a fixed-elevation dock. The plastic samples were deployed at a depth of approximately 0.5 m below surface. For more information regarding the deployment, see Rochman et al. (2013). Upon collection, after deployment, plastic pellets were stored at − 20 °C until analysis.

### Extraction

In total, 26 samples, including blank polymers, were extracted. Samples PVC NMF 9 and PET NMF 12 were not extracted because of insufficient amounts of sample material (Table S1 and Table S2 of the SI). Replicate samples were not available for this study. Samples were weighed into 15 ml brown glass vials and filled up with approximately 10 ml of n-hexane, masses ranged from 0.7 to 2.5 g (Table S1 of the SI). Vials were placed into an ultra-sonication bath for 60 min, and the extracts were then transferred to Erlenmeyer flasks and fresh solvent was added to the remaining samples. The sonication procedure was repeated two times. N-hexane extracts were evaporated with a rotary evaporator to a volume of 1 ml and subsequently split into two aliquots. Pellets with masses equal or less than 1.3 g were not split and those extracts were only used for bioassay evaluations, these samples were PVC SI 9, PVC SI 12, PVC HE 12, and PET HE 12. One aliquot of each extract was further evaporated and solvent exchanged into 25 μl of dimethyl sulfoxide (DMSO) for use in bioassays. The second aliquot was further evaporated and solvent exchanged into 400 μl of toluene for use of quantification of PACs. Toluene extracts were spiked with labeled internal (IS) and recovery (RS) standards, which resulted in 22 extracts for chemical analysis.

### H4IIE-luc assay

Sample PVC HE 9 was not available for bioassay analysis, which reduced the total number of samples tested in the bioassay to 25 (Table S1 of the SI). AhR-mediated potencies of extracts were quantified by use of the H4IIE-*luc* assay, which is a transactivation assay based on a recombinant rat hepatoma cell line, which has been stably transfected with a luciferase reporter gene (Murk et al. 1996). The H4IIE-*luc* assay was performed as previously described (Larsson et al. 2013). Prior to exposure, dilutions of sample extracts were prepared as fourfold serial dilutions of the initial concentration in culture medium. Six different concentrations were derived and added to the test plates. Initial medium concentrations ranged from 245 mg plastic/ml for the sample PVC HE 12 to 540 mg plastic/ml for PET SI 9. On each test plate, a TCDD standard curve of seven concentrations (0.4–300 pM) and a solvent control of DMSO were tested as well. The concentration of DMSO in test media exposure was 0.4%. Samples, standards, and solvent controls were tested in triplicate wells on each plate. After a 24-h incubation, exposure medium in the wells was discarded and the wells were washed twice with 100 μl of phosphate-buffered saline solution (PBS). Cells were lysed in 25 μl of PBS and 25 μl of steadylite plus™ substrate mix and incubated for 15 min in darkness at room temperature for enzymatic reaction to take place. Cell lysates were transferred to white, 96-well, microtiter plates, and the luciferase activity in each well was measured in a luminescence plate reader (FLUOstar Omega; BMG Labtech). Activity of luciferase was expressed in counts per second (CPS) and is proportional to the potency of AhR agonists in the mixture. Concentration-response curves for TCDD and extracts were obtained by use of a sigmoidal concentration-response (variable slope) equation (GraphPad Prism® 5.0 software). The cells were microscopically examined for cytotoxicity before and after the exposure.

### Chemical analysis

Quantification of 42 polycyclic aromatic compounds, including PAHs, oxy-PAHs, and azaarenes, was performed by use of an Agilent 7890A gas chromatograph, coupled to a 5975C, low-resolution mass spectrometer (GC/LRMS), which was equipped with a ZB-SemiVolatiles column (30 m × 0.25 mm, 0.25 μm film thickness; Phenomenex). The initial oven temperature was 90 °C (held for 2 min), ramped by 8 °C min^−1^ to 300 °C (held for 10 min). All measurements were performed in single ion-monitoring mode. Identification and quantification of the PACs in extracts were done by use of quantification mixtures including 24 PAHs and 18 oxy-PAHs and azaarenes in addition to IS and RS. Concentrations of PACs were calculated by use of the isotope dilution method.

### Data analysis

For data analysis of the bioassay results, the raw data was tested for outliers with a Grubbs’ test (alpha = 0.05) using the program QickCalcs outlier calculator by GraphPad Software. Bioassay-derived TCDD equivalents (bio-TEQs) were calculated from concentration-response curves by relating the luciferase induction potency of the samples to that of the TCDD standard as described previously (Larsson et al. 2013). Only plates with a TCDD EC50 value between 6.3 and 14 pM and a maximal induction factor (MIF) between 7 and 14 were used in TEQ calculations. Limit of detection (LOD) in the assay was calculated as the mean of the solvent control (DMSO) plus three times the standard deviation.

Chemically derived TCDD equivalents (chem-TEQs) were calculated as the sum of the product of individual concentrations of PACs multiplied with their H4IIE-luc assay-specific relative potency factors (REP) based on 25% effect concentration (EC25) (Eq. 1). The applied REP values were previously established and published by our laboratory (Larsson et al. 2012, 2014).

1 $$ \mathrm{Chem}-\mathrm{TEQ}\ \left[\mathrm{pg}/\mathrm{g}\right]=\sum {\mathrm{Concentration}}_{\mathrm{compound}}\ \mathrm{x}\ {\mathrm{REP}}_{\mathrm{compound}} $$

The potency balance was calculated as the percentage of the fraction of quantified PACs (chem-TEQ) that can explain the AhR-mediated potency (bio-TEQ).

Statistical analyses were conducted by using GraphPad Prism® 5.0 software, and statistical significance was defined as *p* < 0.05.

## Results

### H4IIE-luc assay

**Fig. 1:**
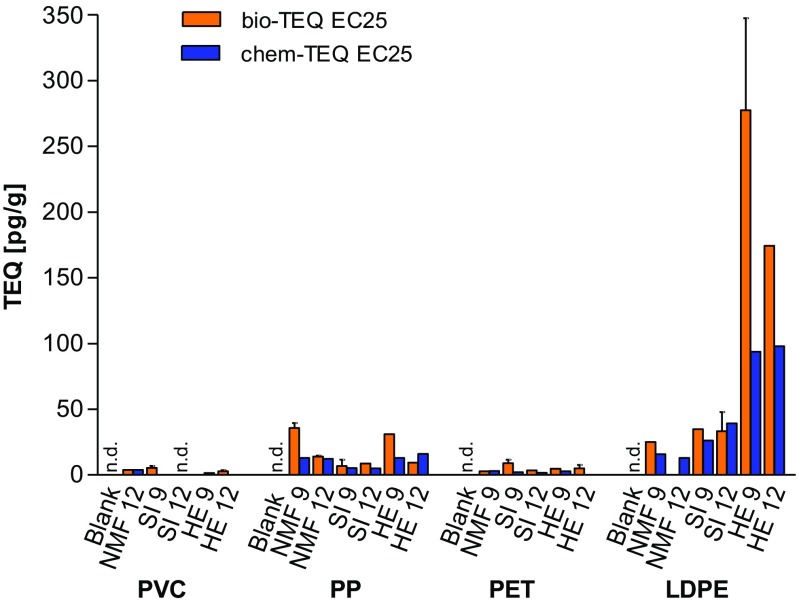
Comparison of concentrations of bio-TEQs and chem-TEQs in pg/g for four preproduction plastic pellets at 9 and 12 months of deployment at three sites determined by use of H4IIE-*luc* assay. Data represent the mean value of n replicates (see Table S2 of the SI) and error bars represent the standard deviation of n replicates. N.d. no detectable response, less than the limit of detection (LOD)

In total, 25 different extracts were tested in the H4IIE-*luc* assay including four blank samples of each polymer type (LDPE, PP, PET, and PVC) before deployment. The blank samples had low AhR-mediated potencies, which were less than the LOD of the assay. Two of the deployed samples also exhibited potencies that were less than the LOD, those were pellets of PVC and PET deployed for 12 months at Shelter Island and Nimitz Marine Facility, respectively. All other samples had quantifiable AhR-mediated potencies in the H4IIE-*luc* assay. Because some of the analyzed samples had efficacies that were less than 50% of the TCDD maximum induction (TMI), bio-TEQs for all samples were calculated at the 25% effect level (Villeneuve et al. 2000). Bio-TEQs ranged from 2.7 pg/g in PET deployed for 9 months at Nimitz Marine Facility (PET NMF 9) to 277 pg/g in low-density PE deployed for 9 months at Harbor Excursion (LDPE HE 9) (Table S2 of the SI). Among the four different polymers, LDPE samples deployed at the sites Harbor Excursion and Shelter Island exhibited greater AhR-mediated potencies (bio-TEQs) at both durations of deployment, compared to the three other polymers (Fig. 1). The differences among plastic types were, however, not significant (ANOVA, *p* = 0.041, followed by Tukey’s test, *p* > 0.05), also the three tested locations were not significantly different from each other (ANOVA, *p* = 0.24). At the third site, the Nimitz Marine facility, PP had the greatest AhR-mediated potency followed by LDPE. Deployed PVC and PET samples exhibited relatively small AhR-mediated potencies with concentrations of bio-TEQ two orders of magnitude less than the greatest concentrations of bio-TEQ in extracts of LDPE. AhR-mediated potency of samples was less in most extracts of samples that had been deployed 12 months compared to those of samples deployed for 9 months (Fig. 1).

### Chemical analysis

**Fig. 2:**
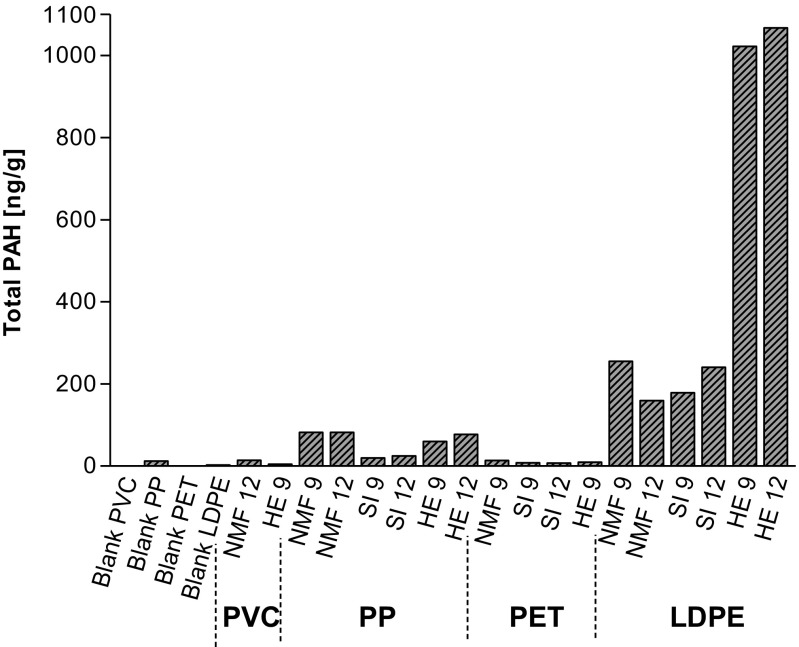
Total concentrations of PAH in ng/g as the sum of 24 PAHs sorbed to four types of plastic pellets deployed for 9 and 12 months at three locations. Blank samples represent the respective preproduction pellet before deployment

Chemical analysis of 24 PAHs, 18 oxy-PAHs, and azaarenes was conducted on 18 deployed plastic pellets and 4 blank preproduction plastic pellets (Table 1). Concentrations of oxy-PAHs and azaarenes were less than the LOD in all samples, while the 24 selected PAHs were detectable in most of the samples. Blank samples contained measurable concentrations and the sum of detected PAHs was greatest in PP (12 ng/g), followed by LDPE (2.1 ng/g) and below LOD in PET and PVC (Table 1). Among the polymers, LDPE sorbed the greatest concentrations of PAHs. Concentrations of the sum of 24 PAHs in deployed samples ranged from 4.6 (PVC HE 9) to 1068 ng/g (LDPE HE 12) (Fig. 2). High molecular weight PAHs with four to five rings were detected in all deployed plastic pellets, including fluoranthene, pyrene, benzo[a]anthracene, chrysene, benzo[b+j]fluoranthene, and benzo[k]fluoranthene (Table 1). The five-ringed PAHs benzo[e]pyrene and benzo[a]pyrene were detected in all deployed samples except for PVC HE 9. The higher molecular weight six-ringed PAH benzo[ghi]perylene was detected in all deployed plastics except for PVC NMF 12. Overall, fluoranthene and pyrene were the most dominant PAHs in the deployed LDPE and PP samples, which are less hydrophobic compared to the other analyzed higher molecular weight PAHs. Among the 24 quantified PAHs, fluoranthene exhibited the greatest concentration of 369 ng/g in low-density PE at Harbor Excursion deployed for 9 months.

**Table 1:** Concentrations (ng/g) of 24 individual PAHs measured in blank and deployed preproduction plastic pellets

PAH/ng g^−1^	LDPE blank	LDPE NMF 9	LDPE NMF 12	LDPE SI 9	LDPE SI 12	LDPE HE 9	LDPE HE 12	PP blank	PP NMF 9	PP NMF 12	PP SI 9	PP SI 12	PP HE 9	PP HE 12	Blank PVC	PVC NMF 12	PVC HE 9	Blank PET	PET NMF 9	PET SI 9	PET SI 12	PET HE 9
Naphthalene	1.6	< 0.4	0.4	< 1.0	0.5	0.6	0.6	0.6	0.3	0.7	0.7	0.3	0.7	0.9	< 0.3	0.7	< 0.4	< 0.3	0.7	< 0.4	0.5	< 0.7
Acenaphtylene	< 0.2	< 0.2	< 0.2	< 0.5	< 0.2	1.0	1.6	< 0.1	< 0.1	< 0.2	< 0.1	< 0.1	< 0.1	< 0.1	< 0.2	< 0.3	< 0.2	< 0.1	< 0.3	< 0.2	< 0.2	< 0.3
Acenaphthene	0.5	0.5	1.9	10	16	11	32	3.7	2.7	2.4	1.4	2.2	2.3	2.3	< 0.3	0.7	< 0.4	< 0.3	< 0.7	0.4	< 0.5	< 0.7
Fluorene	< 4.4	5.6	< 4.9	< 12	4.5	11	11	< 3.1	< 3.3	3.5	< 3.5	3.5	< 3.1	< 3.4	< 3.9	< 8.0	< 4.5	< 3.3	< 8.4	< 4.5	< 6.1	< 8.1
Phenanthrene	< 0.8	12	13	5.1	7.2	56	51	5.7	5.8	6.7	0.7	1.3	4.0	4.9	< 0.2	0.9	0.3	< 0.4	0.5	0.7	0.3	< 0.3
Anthracene	< 0.5	4.2	1.4	1.7	2.3	20	25	0.4	2.5	1.7	0.4	0.6	1.8	2.3	< 0.3	1.1	0.6	< 0.3	0.7	0	0.7	1.1
4H-Cyclopenta[d,e,f]phenanthrene	< 0.2	8.8	5.7	4.5	5.7	24	25	< 0.1	1.0	1.1	0.1	0.1	0.8	0.9	< 0.2	< 0.3	< 0.2	< 0.1	< 0.3	< 0.2	< 0.2	< 0.3
Fluoranthene	< 0.2	95	63	54	63	369	358	1.6	19	22	2.2	2.5	13	16	< 0.2	3.3	0.8	< 0.2	2.6	0.7	0.7	0.7
Pyrene	< 0.2	63	29	35	43	238	245	0.2	11	11	2.0	1.9	8.2	9.5	< 0.2	0.7	0.3	< 0.3	0.9	0.4	0.3	0.4
Benzo[a]fluorene	< 0.2	13	1.9	2.4	2.7	17	18	< 0.1	4.4	1.2	0.2	0.2	1.1	1.5	< 0.2	< 0.3	< 0.2	< 0.1	< 0.3	< 0.2	< 0.2	< 0.3
Benzo[a]anthracene	< 0.3	14	12	6.8	9.2	52	57	< 0.3	5.3	5.5	0.7	0.7	2.8	4.0	< 0.3	1.5	0.5	< 0.3	1.2	0.5	0.5	0.9
Chrysene	< 0.3	14	8.6	10	14	62	66	< 0.3	6.7	6.1	2.0	1.9	3.9	6.2	< 0.3	0.7	0.3	< 0.3	1.4	0.5	0.3	0.7
Benzo[b+j]fluoranthenes	< 0.2	9.4	8.0	22	31	61	69	< 0.1	8.6	8.1	3.6	3.5	7.6	9.9	< 0.2	1.8	1.0	< 0.1	2.1	1.6	1.2	1.3
Benzo[k]fluoranthene	< 0.2	3.8	3.1	5.1	8.0	21	20	< 0.1	2.9	2.5	1.2	1.0	2.7	3.3	< 0.2	1.1	0.4	< 0.2	0.7	0.5	0.3	0.7
Benzo[e]pyrene	< 0.2	5.0	3.5	11	15	32	35	< 0.1	3.2	2.5	1.5	1.4	3.1	4.4	< 0.2	0.7	< 0.2	< 0.1	1.2	0.7	0.8	0.9
Benzo[a]pyrene	< 0.2	4.4	4.1	3.4	5.7	24	26	< 0.1	4.5	3.8	1.6	1.6	3.6	4.8	< 0.2	0.7	< 0.2	< 0.1	0.5	0.6	0.5	0.9
Perylene	< 0.2	1.1	1.1	< 0.5	1.5	5.7	5.7	< 0.1	0.7	0.2	< 0.1	0.1	0.5	0.3	< 0.2	< 0.3	< 0.2	< 0.1	< 0.3	< 0.2	< 0.2	< 0.3
Indeno[1,2,3-cd]pyrene	< 0.2	1.1	1.4	3.1	5.5	8.0	9.4	< 0.1	1.9	1.4	1.0	1.3	1.9	2.7	< 0.2	0.4	0.3	< 0.1	0.5	< 0.2	0.5	0.7
Dibenzo[aj]anthracene	< 0.2	< 0.2	< 0.2	< 0.5	1.1	1.7	2.0	< 0.1	0.5	0.4	< 0.1	< 0.1	0.4	0.6	< 0.2	< 0.3	< 0.2	< 0.1	< 0.3	< 0.2	< 0.2	< 0.3
Dibenzo[ac+ah]anthracenes	< 0.2	< 0.3	< 0.3	< 0.7	< 0.2	1.4	1.7	< 0.2	< 0.2	0.6	< 0.2	< 0.2	0.6	0.7	< 0.2	< 0.5	< 0.3	< 0.2	< 0.5	< 0.3	< 0.2	< 0.5
Benzo[ghi]perylene	< 0.2	0.9	1.1	3.1	4.9	7.2	8.9	< 0.1	1.4	0.6	0.7	0.7	0.8	1.9	< 0.2	< 0.3	0.1	< 0.1	0.7	0.7	0.3	0.7
Naphtho[2,3-a]pyrene	< 0.2	< 0.3	< 0.2	< 0.6	< 0.2	< 0.3	< 0.3	< 0.2	< 0.2	< 0.2	< 0.2	0.2	< 0.2	< 0.2	< 0.2	< 0.4	< 0.2	< 0.2	< 0.4	< 0.2	< 0.2	< 0.4
∑ 24 PAHs	2.1	256	159	177	241	1024	1068	12	82	82	20	25	60	77	0.0	14	4.6	0.0	14	7.3	6.9	9.0

All calculated chem-TEQ values for blank samples were less than the LOD. Chem-TEQ values ranged from 1.5 (PVC HE 9) to 98 pg/g (LDPE HE 12) (Table S2). The greatest chem-TEQ values were calculated for LDPE compared to the three other plastic pellets, followed by PP, PET, and PVC with the lowest calculated chem-TEQ. Potency balances were between 24 and 170% (Table S2).

## Discussion

The results of this study present experimental evidence to corroborate the hypothesis by Rochman et al. (2013) that PE plastics might pose a greater toxicological risk compared to PP, PET, and PVC. In particular, the finding that LDPE had the greatest AhR-mediated potencies compared to the other tested polymers was expected, considering the fact that polyethylene has been recognized as a good sorbent for HOCs in passive sampling devices (Zabiegala et al. 2010). This is of importance since PE is a high-volume production polymer used in, for example, packaging materials (PlasticsEurope 2016) and is frequently found in microplastic fractions in surface waters globally (Hidalgo-Ruz et al. 2012). Interesting for a toxicological assessment of plastic in the marine environment is the fact that six out of eight pairs of the samples were less potent in activating the AhR pathway after 12 months of deployment compared to 9 months. This was independent of the type of polymer but most pronounced in LDPE at Harbor Excursion. The decline in AhR-mediated potency at 12 months illustrated by bioassay results cannot be attributed to a possible desorption and/or degradation of the analyzed PAHs, because most samples illustrated a greater total concentration of PAHs after 12 months compared to 9 months. In the study by Rochman et al. (2013), using more time data points, total concentrations of PAHs adsorbed to LDPE and PP were found to be in equilibrium at 6 months and for PET and PVC equilibrium occurred at 3 months. AhR ligands other than the quantified PAHs could have been desorbed from the plastic pellets or degraded during the period of deployment from 9 to 12 months. Biofilms with a diverse microbial community, such as hydrocarbon-degrading bacteria, can develop on marine plastic and possibly biodegrade the plastic polymer (Zettler et al. 2013). Hence, a reduction of Ah receptor agonists sorbed to the surface of the polymer, other than quantified PAHs and PCBs, might occur and lead to a decreased induction in the bioassay after a longer time of deployment. Therefore, preproduction plastic pellets might potentially become less toxic with time in the aquatic system due to biodegradation. It has been reported from passive sampling devices that fouling can lead to a decrease in the uptake of HOCs compared to not fouled devices (Harman et al. 2009). A longer residence time in the marine environment might also lead to an increased leaching of unreacted monomers and additives from the plastic products to the surrounding environment, which could lead to a lesser AhR-mediated potency in the bioassay when they are AhR agonists. It is also possible that other chemicals, which act as AhR antagonists, sorbed to the pellets with continuing time. These antagonists could have been masking the effects of the Ah receptor agonists and therefore may have led to a decrease in potency in the bioassay from 9 to 12 months. For instance, copper that has been found as a pollutant of concern in the San Diego Bay marina (CA.EPA 2016) could have been sorbed to the preproduction pellets as it has been shown to be present in marine plastics (Ashton et al. 2010; Holmes et al. 2012), and can lead to an inhibition of AhR expression (Darwish et al. 2014; Korashy and El-Kadi 2004). The ability to conclude that the AhR-mediated potency decreased with continuing residence time in the marine system was hampered by a limited sample amount of some samples, resulting in the inability to analyze all time points (see Table S2 in the supporting information). The study design by Rochman et al. (2013) included two replicates for each time point and sampling site. Replicate samples were not available for analysis in the present study, but no significant variability in concentrations of PAHs and PCBs among the replicates could be observed by Rochman et al. (2013).

For a better understanding of the potential impact of dioxin-like contaminants adsorbed to marine plastic debris, it is important to investigate whether the finding that AhR-mediated potency decreased with extended marine residence time can be applied to even longer weathered microplastics as well. This is particularly of relevance because chemicals with a greater hydrophobicity and higher molecular weight are expected to reach equilibrium later than the lower molecular weight and less hydrophobic chemicals (Muller et al. 2001). It has been estimated that sorption of most HOCs to marine microplastics with a size of 0.5–5 mm will be at equilibrium after 2 years (Koelmans et al. 2016). There is one study so far that demonstrated that weathered plastic pellets had greater distribution coefficients for phenanthrene compared to preproduction PE and PP samples, however, these materials had slower diffusion kinetics (Karapanagioti and Klontza 2008). This would suggest that weathered plastics are able to bind a greater amount of pollutants over time, and thus could lead to greater AhR-mediated potencies than their primary plastic counterparts. The sorption should be further investigated, however, testing for different types of polymers, different pollutants with a range of log K_ow_ values, and importantly for different particle sizes. It has been shown in a model that with decreasing pellet diameter, the sorption of pollutants to plastic pellets will be faster (Endo et al. 2013). Since weathered plastics are more prone to break down into smaller pieces due to embrittlement, those particles might absorb pollutants even faster than their preproduction counterparts.

The quantification of 24 PAHs is consistent with previous findings that reported LDPE to have the highest sorption capacity, followed by PP, PET, and PVC (Rochman et al. 2013). San Diego Bay is a natural harbor and deep-water port with facilities for cargo and cruise ship terminals. The adjacent areas of the bay are highly urbanized and the San Diego International airport is in close proximity to the sampling site Harbor Island. The two other sampling sites are influenced by several recreational marinas as well as commercial fishing fleets. Therefore, it was expected to find chemicals of anthropogenic origin in the plastic samples, especially PAHs from pyrogenic sources formed by incomplete combustion of fossil fuels, e.g., from ships, as was demonstrated in a previous study by Rochman et al. (2013). The prevalence of PAHs was reflected in the present study by the relatively great contribution of PAHs to the AhR-mediated potency in the samples. Fluoranthene was observed to be major component of the total PAH concentrations in all deployed LDPE samples and most of the PP samples. This is in accordance with surface sediments from San Diego marinas in which the higher molecular weight PAHs (4–6 rings) were detected as the major contributors to total PAH concentrations of 36 individual PAHs (Neira et al. 2017). However, for the assessment of AhR-mediated signaling, fluoranthene is negligible because this compound does not activate the Ah receptor following a 24-h exposure (Larsson et al. 2012). In all analyzed PET and PVC samples, fluorene was the main contributor to the sum of the concentrations of individual PAHs. Fluorene is a lower molecular weight PAH and a weak AhR agonist that does not contribute to the AhR-mediated potency in the bioassay, which was also reflected by the low bio-TEQ results for these polymers. In all deployed samples, benzo[b+j]fluoranthenes and benzo[k]fluoranthene were the major contributors to the overall biological efficacy.

The quantified PAHs explained more than 50% of the observed AhR-mediated potency in 12 of the 16 samples for which both bio-TEQ and chem-TEQ values were available. However, in samples with the greatest potential for AhR pathway activation (LDPE HE 9 and LDPE HE 12), there is an unexplained potency of 66% and 44%, respectively. Many other compounds are able to bind to the AhR and activate the AhR pathway including PCBs, polychlorinated dibenzo-p-dioxins (PCDDs), and polychlorinated dibenzofurans (PCDFs) (Lee et al. 2013; Van den Berg et al. 2006). In fact, the presence of other pollutants besides PAHs have been reported in the San Diego Bay, such as PCBs, copper, mercury, zinc, chlordane, and lindane/hexachlorocyclohexane (HCH) (CA.EPA 2016). The previous study by Rochman et al. (2013) observed measurable levels of PCBs in all of the plastic samples with the greatest PCB concentrations at the sampling site Shelter Island. At this site, LDPE did not reach equilibrium by 12 months, while PET and PVC reached equilibrium by 6 months and PP was at equilibrium at 12 months. The time to reach equilibrium for the total concentration of PCBs sorbed to LDPE at Shelter Island was estimated to be 19 months. Although the total PCB concentrations were noticed to be approximately one to two orders of magnitude less than the measured total PAH concentrations, some PCBs are more potent AhR agonists than PAHs. They are, therefore, able to induce a greater potency for the receptor in a mechanism specific bioassay such as the H4IIE-*luc*. Reported H4IIE-*luc*-specific REP values for 11 PCBs (CB#81, 77, 123, 118, 114, 105, 126, 167, 156, 169, and 189) ranged from 1 × 10^−9^ to 0.1 (Lee et al. 2013). PCB 126 was observed to have the greatest REP value of 0.1 in the H4IIE-*luc* assay (Lee et al. 2013), whereas the most potent PAH analyzed in the present study, benzo[k]fluoranthene, had a REP value of 0.002 (Larsson et al. 2012). In order to determine a possible contribution of PCBs to the measured bio-TEQs, theoretical chem-TEQ values were calculated for the polymers LDPE, PP, and PET, based on the previously reported concentrations of individual PCB congeners by Rochman et al. (2013) and the reported REP values by Lee et al. (2013). Overall, it was observed that PCBs did not contribute to the measured bio-TEQs, except in one of the duplicate LDPE SI 9 samples where PCBs contributed about 3% to the bio-TEQ. Considering the theoretically calculated contribution of PCBs and PAHs to the AhR-mediated potency in the samples, results suggest that the remaining unexplained potency in the bioassay must be attributed to AhR-activating compounds other than the measured PAHs and PCBs. Dioxins, for example, are more potent AhR agonists than PAHs (Van den Berg et al. 2006). An effect-directed analysis could be a useful tool to implement here in order to identify causative agents.

Because the bioassay is integrating the complete potency of chemicals present in a mixture and chemical analysis is only looking for target compounds, it is possible that given only the chem-TEQs, one would over- or underestimate the potency in the bioassay (Table S2). It is possible that the chemicals are interacting with each other in a way that is masking the effect of quantified PAHs present in the mixture, which is reflected in a greater chem-TEQ than bio-TEQ. On the other hand, the chemicals can maybe have additive effects (Giesy and Kannan 1998), and thus elicit a greater bio-TEQ compared to chem-TEQ.

As this is the first study to measure AhR-mediated potency on microplastics deployed in the marine system there is no comparable bioassay data available. Nevertheless, there is data available from sediment toxicity studies. One study of sediment samples from the estuary at the river Elbe in Germany revealed bio-TEQ values ranging from 45 pg/g dry matter (d.m.) to 268 pg/g d.m., determined by use of the H4IIE-*luc* assay (Otte et al. 2013). These values are similar to the bio-TEQs for the deployed plastic pellets. PAH concentrations of the 16 priority PAHs defined by the US EPA were reported to range from < 20 to 906 ng/g d.m., which is comparable to concentrations found in the present study and underlines the role of plastic pellets as a sorbent for environmental pollutants, such as PAHs, with similar sorptive capacities as sediment.

A study on surface sediments in San Diego Bay marinas revealed concentrations of the 16 priority PAHs to range from 246 to 3452 ng/g d.m. sediment at sampling sites in Shelter Island and 606–19,967 ng/g d.m. at sampling sites in Harbor Excursion East (Neira et al. 2017). The LDPE samples at Harbor Excursion were observed to be within this range with a total 16 priority PAH concentration of 943 and 982 ng/g at 9 and 12 months, respectively. Concentrations of LDPE samples from Shelter Island were found to be less than concentrations of sediment samples, with concentrations of 160 and 195 ng/g by 9 and 12 months. However, PAH concentrations have been shown to vary about one or two orders of magnitude at different sampling sites in the same marina (Neira et al. 2017) resulting in large spatial variation at the same sampling site.

Assessing the risk of AhR-mediated effects due to microplastic exposure is not possible since the intake and bioavailability of AhR agonists from microplastics is unknown. Concentrations of total PAHs in this study, however, were much less than the effect range-low (ERL) of 4022 ng/g d.m. for sediments, established by the US National Oceanic and Atmospheric Administration (NOAA) (Buchman 2008). Even when considering only the16 priority PAHs, the ERL (2968 ng/g) is almost three times that of the greatest total PAH concentration of 1068 ng/g (LDPE HE 12) detected in this study.

Due to the fact that an exhaustive extraction method was used in this study, a worst case scenario of desorption of pollutants from the plastic pellets was represented. It might be unlikely that the same amount of pollutants would desorb within an aquatic organism from microplastics of the size used in this study during the residence time in the body of an organism, however, this needs to be confirmed with experimental data. Because some sites in the San Diego Bay marina were classified as potentially toxic for benthic organisms (Neira et al. 2017), the body burden of the resident organisms can be expected to be already high, and ingested plastic might not lead to elevated levels of organic pollutants in the bodies. For example, it has been demonstrated that the transfer of pollutants into tissues of lugworms can be higher from sand compared to microplastics (Browne et al. 2013). Therefore, uptake of pollutants from other marine matrices, such as sediments, colloids, and dissolved organic carbon (DOC), might predominate because they are more abundant in most habitats than plastic and also hold a greater percentage of sorbed HOCs than plastic (Koelmans et al. 2016). Additionally, prey as a source of pollutants needs to be considered. Nevertheless, marine plastic is able to travel over long distances and can be transported into less polluted areas. Therefore, it might have a bigger impact on organisms that live in these habitats than in already polluted sites. Furthermore, it has to be stressed that the AhR-mediated potency is given on a per 1 g of plastic basis, which is, for example, ten times greater than the ecological quality objective (EcoQO) of < 0.1 g ingested plastic that should be achieved in 10% of the seabird population of northern fulmars (OSPAR 2008). It might not be the case in a common marine environment that 1 g of plastic will be ingested by an organism, but it could be the case for accumulation zones and other hot spots of plastics. Unfortunately, a vast number of publications report ingested plastic pieces only as number of pieces and do not give the weight or diameter of those pieces. This information could be beneficial in the future to estimate the load of chemicals that can be attached to ingested plastics and ultimately might be able to transfer into marine organisms.

## Conclusions

The measurable in vitro AhR-mediated potencies (bio-TEQs) in preproduction plastic pellets, of which 24 selected PAHs contributed more than 50% to the bio-TEQ in 76% of the samples, varied by the type of polymer. Extracts of LDPE demonstrated the greatest potential for AhR pathway activation as well as greatest sorption capacities of PAHs. This reinforces the hypothesis that PE has the potential to hold a greater toxicological risk than PP, PET, and PVC. The background levels of AhR-mediated potencies of marine microplastics are unknown and should be investigated in remote areas. The observed tendency for a decreased AhR-mediated potency in some of the 12-month deployed samples compared to 9 months should be further investigated and verified in another set of field deployed samples. In the future, other mechanisms of action such as endocrine receptor-mediated pathways should be assessed as well in order to get a more complete understanding of the possible impacts of marine microplastics.

## Electronic supplementary material

ESM 1 (DOCX 76 kb)
