# Supplementary material for: Aryl hydrocarbon receptor-mediated potencies in field-deployed plastics vary by type of polymer
Source: Environ Sci Pollut Res Int. 2019 Feb 4;26(9):9079–88. doi: 10.1007/s11356-019-04281-4 (PMC6469617; doi:10.1007/s11356-019-04281-4)
Supplement: Supplementary file 1 — (DOCX 76 kb) [file 11356_2019_4281_MOESM1_ESM.docx]

**Supporting Information**

**Materials and Methods**

***Chemicals***

An internal standard (IS) mixture, PAH-mix 31, containing D8-naphthalene, D10-acenaphthene, D10-phenanthrene, D12-chrysene and D12-perylene and a recovery standard (RS) D10-fluoranthene (99 - 99.5 % purity) were purchased from Labor; Dr. Ehrenstorfer-Schärfers (Augsburg, Germany). D8-anthraquinone was purchased from Chiron AS (Trondheim, Norway). A PAH standard mixture containing 16 PAHs (Mix 63): naphthalene, acenaphthene, acenaphthylene, anthracene, phenanthrene, pyrene, fluorene, fluoranthene, benzo[a]pyrene, indeno[1,2,3-cd]pyrene, benzo[b]fluoranthene, chrysene, dibenzo[a,h]anthracene, benzo[g,h,i]perylene, benzo[k]fluoranthene and benzo[a]anthracene (98-99.50% purity) was purchased from Labor; Dr. Ehrenstorfer-Schärfers. Cyclopenta[d,e,f]phenathrene (97 %) was purchased from Sigma-Aldrich (Stockholm, Sweden) and benzo[a]fluorene (98 %), naphtho[2,3-a]pyrene (99 %) and benzo[j]fluoranthene (99 %) were purchased from Ultra Scientific Analytical Solutions (North Kingstown, USA). Dibenzo[a,c]anthracene (99.8 %), perylene (99 %), benzo[e]pyrene (99 %) and dibenzo[a,j]anthracene (99.8 %) were purchased from Labor; Dr. Ehrenstorfer-Schärfers. 9-fluorenone (98 %), naphthacene-5,12-dione (97 %), 9,10-dihydrobenzo[a]pyren-7(8H)-one (97 %), quinoline (98 %), carbazole (99.3 %), anthracene-9,10-dione (99. 8%), 4Hcyclopenta[d,e,f]phenanthrenone (BCR-338; 99.5 %), benzo[a]fluorenone (BCR-342; 99.8 %), and 6H-benzo[cd]pyren-6-one (BCR-339; 98.8 %) were purchased from Sigma-Aldrich, 1-indanone (>99 %), 2-methylanthracene-9,10-dione (97 %), benzo[a]anthracene-7,12-dione (>98 %), 7H-benzo[de]anthracene-7-one (99 %), benzo[h]quinoline (98 %), and acridine (> 98 %) were purchased from Alfa Aesar (Karlsruhe, Germany), 1,4-chrysenequinone (>93 %) was obtained from Tokyo Chemical Industry (Zwijndrecht, Belgium) and dibenzo[ah]acridine (99.6 %) was purchased from LGC standards.

***Deployment***


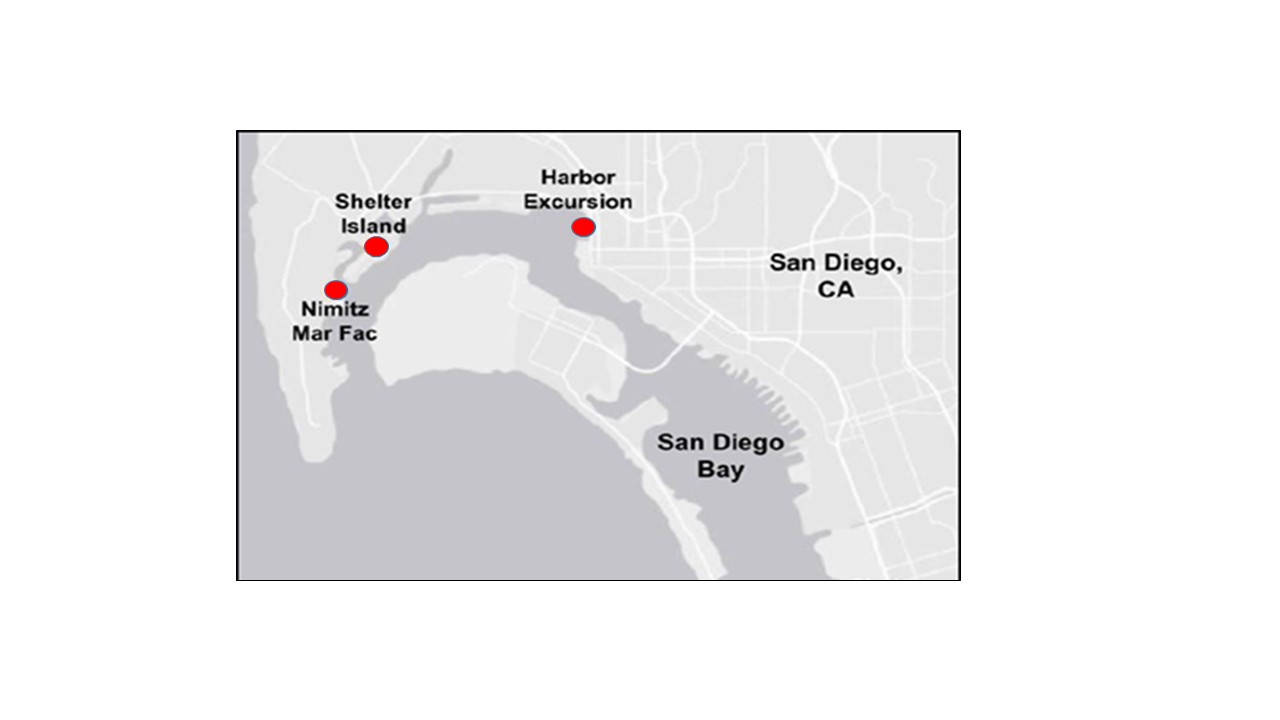


**Figure S1**. Map of San Diego Bay, showing locations where samples were deployed near the Nimitz Marine Facility, Shelter Island and the Harbor Excursion pier (taken from Rochman et al. (2013) and modified).

***Extraction***

**Table S1.** Preproduction plastic pellets used for extraction with n-hexane and tested samples in chemical and bioassay analysis.

| **Sample** | **Weight of pellets for extraction [g]** | **Tested in bioassay (n = 25)** | **Chemical analysis (n = 22)** |
| --- | --- | --- | --- |
| Blank PVC | 2.5 | X | X |
| PVC NMF 9 | / | / | / |
| PVC NMF 12 | 1.4 | X | X |
| PVC SI 9 | 0.9 | X | - |
| PVC SI 12 | 1.1 | X | - |
| PVC HE 9 | 1.7 | / | X |
| PVC HE 12 | 0.7 | X | - |
| Blank PP | 2.2 | X | X |
| PP NMF 9 | 2.2 | X | X |
| PP NMF 12 | 2.2 | X | X |
| PP SI 9 | 2.0 | X | X |
| PP SI 12 | 2.0 | X | X |
| PP HE 9 | 2.3 | X | X |
| PP HE 12 | 2.2 | X | X |
| Blank PET | 2.1 | X | X |
| PET NMF 9 | 1.7 | X | X |
| PET NMF 12 | / | / | / |
| PET SI 9 | 2.5 | X | X |
| PET SI 12 | 2.0 | X | X |
| PET HE 9 | 2.1 | X | X |
| PET HE 12 | 1.3 | X | - |
| Blank LDPE | 2.0 | X | X |
| LDPE NMF 9 | 2.1 | X | X |
| LDPE NMF 12 | 1.9 | X | X |
| LDPE SI 9 | 1.5 | X | X |
| LDPE SI 12 | 2.0 | X | X |
| LDPE HE 9 | 2.2 | X | X |
| LDPE HE 12 | 1.9 | X | X |

n = number of samples tested; X = tested sample; / = sample not available; - = weight of sample equal or less than 1.3 g, therefore only tested in bioassay

***Chemical analysis***

Toluene spiked with internal and recovery standard was used as an instrumental blank sample in order to establish a LOD value for each compound. The concentrations of the sum of 24 PAHs are given as the sum of individual concentrations excluding the values that were less than the LOD. Chem-TEQ values were calculated based on PAH concentrations that were above the LOD.

**Results**

**Table S2.** Overview of calculated bio-TEQs and chem-TEQs for four polymers deployed at three locations for 9 or 12 months and the calculated potency balance. Bio-TEQs were derived from an effective concentration of 25 % (EC25) in the H4IIE-*luc* assay. Chem-TEQs were calculated from individual PAH concentrations multiplied by corresponding individual PAH relative potency factors (REPs) at an effective level of 25 % (EC25) in the H4IIE-*luc* assay.

| **Sample** | **n_H4IIE-_*_luc_*** | **bio-TEQ_EC25_**  **[pg/g]** | **chem-TEQ_EC25_ [pg/g]** | **Potency balance [%]** |
| --- | --- | --- | --- | --- |
| Blank PVC | 1 | n.d. | <0.1 |  |
| PVC NMF 9 |  | n.s. | n.s. |  |
| PVC NMF 12 | 1 | 3.8 | 3.8 | 100 |
| PVC SI 9 | 2 | 5.4 | n.a. |  |
| PVC SI 12 | 1 | n.d. | n.a. |  |
| PVC HE 9 |  | n.a. | 1.5 |  |
| PVC HE 12 | 2 | 2.9 | n.a. |  |
| Blank PP | 1 | n.d. | <0.1 |  |
| PP NMF 9 | 2 | 36 | 13 | 36 |
| PP NMF 12 | 2 | 14 | 12 | 86 |
| PP SI 9 | 2 | 6.6 | 5.2 | 79 |
| PP SI 12 | 1 | 8.8 | 4.9 | 56 |
| PP HE 9 | 1 | 31 | 13 | 42 |
| PP HE 12 | 1 | 9.4 | 16 | 170 |
| Blank PET | 1 | n.d. | <0.1 |  |
| PET NMF 9 | 1 | 2.7 | 3.0 | 111 |
| PET NMF 12 |  | n.s. | n.s. |  |
| PET SI 9 | 2 | 8.9 | 2.1 | 24 |
| PET SI 12 | 1 | 3.3 | 1.7 | 51 |
| PET HE 9 | 2 | 4.7 | 2.7 | 57 |
| PET HE 12 | 2 | 5.0 | n.a. |  |
| Blank LDPE | 1 | n.d. | <0.1 |  |
| LDPE NMF 9 | 1 | 25 | 16 | 64 |
| LDPE NMF 12 | 0 | n.v. | 13 |  |
| LDPE SI 9 | 1 | 35 | 26 | 74 |
| LDPE SI 12 | 2 | 33 | 39 | 118 |
| LDPE HE 9 | 2 | 277 | 94 | 34 |
| LDPE HE 12 | 1 | 174 | 98 | 56 |

n_H4IIE-_*_luc_*= number of independent replicates within the validity criteria; n.d. = no detectable response; n.a. = not analyzed; n.v. = not valid according to the bioassay quality criteria; n.s. = no sample

**References**

Rochman CM, Hoh E, Hentschel BT, Kaye S. 2013. Long-term field measurement of sorption of organic contaminants to five types of plastic pellets: Implications for plastic marine debris. Environ Sci Technol 47:1646-1654.
